# Exploring individual differences in musical rhythm and grammar skills in school-aged children with typically developing language

**DOI:** 10.1038/s41598-022-21902-0

**Published:** 2023-02-07

**Authors:** Rachana Nitin, Daniel E. Gustavson, Allison S. Aaron, Olivia A. Boorom, Catherine T. Bush, Natalie Wiens, Chloe Vaughan, Valentina Persici, Scott D. Blain, Uma Soman, David Z. Hambrick, Stephen M. Camarata, J. Devin McAuley, Reyna L. Gordon

**Affiliations:** 1 Vanderbilt Brain Institute, Vanderbilt University, Nashville, TN USA; 2 Department of Otolaryngology - Head & Neck Surgery, Vanderbilt University Medical Center, Nashville, TN USA; 3 Department of Medicine, Division of Genetic Medicine, Vanderbilt University Medical Center, Nashville, TN USA; 4 Vanderbilt Genetics Institute, Vanderbilt University Medical Center, Nashville, TN USA; 5 Institute for Behavioural Genetics, University of Colorado Boulder, Boulder, CO USA; 6 Department of Speech, Language and Hearing Sciences, Boston University, Boston, MA USA; 7 Department of Hearing and Speech Sciences, Vanderbilt University Medical Center, Nashville, TN USA; 8 Department of Speech-Language-Hearing: Sciences and Disorders, University of Kansas, Lawrence, KS USA; 9 Ascension Via Christi St Teresa Hospital, Wichita, KS USA; 10 Department of Human Sciences, University of Verona, Verona, Italy; 11 Department of Psychology, Università degli Studi di Milano - Bicocca, Milan, Italy; 12 Department of Psychiatry, University of Michigan-Ann Arbor, Ann Arbor, MI USA; 13 Department of Communication Disorders and Deaf Education, Fontbonne University, St. Louis, MO USA; 14 Department of Psychology, Michigan State University, East Lansing, MI USA; 15 Department of Psychology, Vanderbilt University, Nashville, TN USA; 16 Vanderbilt Kennedy Center, Vanderbilt University Medical Center, Nashville, TN USA

**Keywords:** Human behaviour, Language, Learning and memory

## Abstract

A growing number of studies have shown a connection between rhythmic processing and language skill. It has been proposed that domain-general rhythm abilities might help children to tap into the rhythm of speech (prosody), cueing them to prosodic markers of grammatical (syntactic) information during language acquisition, thus underlying the observed correlations between rhythm and language. Working memory processes common to task demands for musical rhythm discrimination and spoken language paradigms are another possible source of individual variance observed in musical rhythm and language abilities. To investigate the nature of the relationship between musical rhythm and expressive grammar skills, we adopted an individual differences approach in N = 132 elementary school-aged children ages 5–7, with typical language development, and investigated prosodic perception and working memory skills as possible mediators. Aligning with the literature, musical rhythm was correlated with expressive grammar performance (r = 0.41, *p* < 0.001). Moreover, musical rhythm predicted mastery of complex syntax items (r = 0.26, *p* = 0.003), suggesting a privileged role of hierarchical processing shared between musical rhythm processing and children’s acquisition of complex syntactic structures. These relationships between rhythm and grammatical skills were not mediated by prosodic perception, working memory, or non-verbal IQ; instead, we uncovered a robust direct effect of musical rhythm perception on grammatical task performance. Future work should focus on possible biological endophenotypes and genetic influences underlying this relationship.

## Introduction

There are a growing number of studies showing correlations between musical rhythm perception and speech/language skills, including grammar^1–4^. Gordon et al.^3^ demonstrated a positive association in children (age 6), such that those who were more accurate at distinguishing between musical rhythm sequences, also tended to have stronger performance on a morpho-syntactic language production task, despite obvious differences in task demands and musical and language stimuli. This type of finding was extended to other grammatical tasks by several groups, including Swaminathan and Schellenberg^4^ who showed a strong association between musical rhythm perception and receptive grammar (with a larger sample size of ~ 100 school-aged children) and Politimou et al.^1^, who showed that rhythm perception was associated with performance on a sentence imitation task (which encompasses both receptive and expressive dimensions of grammatical skill) in pre-schoolers. Moreover, Lee et al.^2^ also found a correlation between rhythm discrimination and receptive grammar in a wider age range of participants (7–17-year-olds), while controlling for auditory working memory and musical training. The goal of this paper was to test the robustness of the relationship between musical rhythm perception and grammatical performance, and then to look at the processes that might mediate this interaction.

Further behavioural investigations into the relationship between rhythm and grammatical skills are important because such studies could provide insight into mediating mechanisms, which in turn can inform us about overlap between how music and language are processed and decoded in the developing brain. An understanding of this relationship is also important in the context of atypical language development. Disorders that affect the use of language and hamper communication have long-term negative impacts on quality of life, economic outcomes, and mental health^5,6^. Children with developmental disorders of reading and spoken language (e.g., developmental dyslexia, developmental language disorder, stuttering) are known to struggle with rhythmic tasks (like rhythm production, discrimination, and synchronisation)^7–9^. A recent synthesis of over 300 studies suggested that atypical (impaired) rhythm is a risk factor for atypical language development^10^; moreover, there is still much to learn about how developmental cascades affected by rhythm processing unfold during development (see also Lense et al.^11^). Therefore, advancing the understanding of the role of rhythm in acquisition of grammatical skills might be instrumental in providing new perspectives into studying language disorders. It is interesting to note that rhythmic priming experiments, in which participants actively listen to musical sequences with a strong and predictable rhythm (prime) prior to language trials (target), have shown that listening to regular rhythms enhances the performance of the listener in a sentence imitation task that measures phonological accuracy^12^, and in grammaticality judgement tasks^13–15^. These effects have been shown in both typically developing participants and in those with reading, hearing, and language disorders^12,14,16^. Taken together, the convergence of evidence of associations between rhythm and grammatical processing at both the *trait* (stable correlations between over time) and *state* (transient improvement in task performance) levels provides further corroboration for overlapping processing of musical rhythm and linguistic grammar.

Such groundwork demonstrating an association between rhythm and grammar task performance, in typical and atypical populations, has led to the question of what underlying cognitive and biological mechanisms might account for such a relationship. In order to dissect the relationship between rhythm and grammar, the current study had two goals: first, we evaluated the associations between music rhythm perception and expressive grammar skills; and second, we examined the role of working memory and prosodic perception as potential mediators of the association between rhythm and grammar. We made use of structural equation modelling so that we could study the direct effect of musical rhythm perception on expressive grammar, the indirect mediation either through prosodic perception or working memory, and because this approach would also help us identify which predictors would have the strongest effect on grammatical ability. We also considered the role of IQ in the structural equation model along with our mediators. In addition to an overall measure of expressive language, we used a complex syntax sub-score from the grammar assessment, given prior associations between rhythm and this subscore^17^. Complex syntax refers to sentences constructed using multiple clauses, that can either be linked with connectors or be embedded^18^. When parsing such sentences, children appear to make use of prosodic cues to facilitate processing of multiple clauses^19–21^. Since prosodic perception is measured in our study, using complex syntax as an additional outcome would be another way to understand its specific role in mediating the rhythm-grammar link.

The first mediator we considered is prosodic perception. In music, rhythm is represented by temporal patterns of sounds organised around the “beat” or pulse of a piece of music, while in language, slow temporal cues are captured by speech prosody, which encompasses intonational and stress patterns of syllables, the lengthening and pausing during and of phrases, and the loudness and pitch of speech^22–24^. These patterns are perceived as the rhythmic components of speech, though prosody may not necessarily adhere to the isochronous meter we tend to experience in musical rhythm^25,26^. Prosody plays a crucial role in early language acquisition and lexical development: for example, prosodic boundaries, which in sentences generally reflect the sentences’ syntactic structure, were shown to be leveraged by toddlers to learn the syntactic function of novel words^27,28^. Five-month-old children can distinguish between their native language and a language from a different rhythmic class, but not between two languages that follow similar rhythmic classes^29^. Studies have also shown that the prosody of sentences affects grammatical processing^30^ and facilitates word learning in toddlers^28^. Sensitivity to prosody is thus important for syntactic parsing and lexical access, in addition to its well-documented roles in decoding nuanced emotions, tone, context, emphasis and semantics^23,27,31,32^. Individual differences in prosodic sensitivity have also been shown to be related to musical rhythm perception as well as with musical training^4,33–35^. Torppa et al.^36^ found that children who are musically engaged have improved performance on tasks that measure stress pattern awareness in speech. The sensitivity to prosodic cues of grammatical structures could be affected by a domain-general rhythm perception ability, since speech and music both require the listener to process hierarchically organised temporal sequences^37^. Such an attunement to rhythm might give some children an advantage in accessing the syntactical nature of grammar, thus allowing prosody to play a mediatory role in how rhythm and grammar are processed and acquired.

The other mediator that we explored is working memory. Working memory is a multi-component system that is primarily employed during task/goal-oriented activities and involves storage of relevant information (verbal or non-verbal), and cognitive manipulation and processing of the stored information to complete said task^38^. When recruited, working memory can also transfer information received during the task to long-term storage, and thus plays an important role in learning^39^. Working memory ability is correlated with performance on rhythmic synchronisation tasks^40^, prosodic perception^41^, ability to distinguish between prosodically deviant sentences^42^, and also with sensitivity to morphosyntactic and grammatical violations^43,44^. Further, just as for prosody, musical ability and training have been shown to correlate with higher working memory spans^45,46^. Another process that is closely related to working memory is cognitive flexibility, which is the ability to effectively switch mental representation or task sets as new information becomes available. Cognitive flexibility might play a role in parsing multi-clause sentences, and has been shown to be positively correlated with beat perception^47^ and as being predictive of developmental dyslexia^48^. In summary, given that working memory (and related processes) are associated with rhythm, grammar, and prosodic abilities, we hypothesised that it could play a mediatory role between rhythm and grammar processing and is thus included in our analysis, implemented with structural equation modelling (path model) in a relatively sizable developmental sample (N = 132 children with typical language development, ages 5–7 years old).

## Results

### Sample characteristics

We screened 150 participants for this study, from which N = 132 (aged 5.1–8.2, mean = 6.5 years, SD = 10 months, 76 females) met the complete eligibility criteria (based on their assessment scores and holistic review of screening measures by Speech-Language Pathologists [SLPs]) and constitute the final dataset (see Methods and Supplementary Information for details). Eligibility for the current study was dependent on participants having normal hearing, typical non-verbal cognitive ability, typical language development, an absence of major neurodevelopmental disorders or conditions (e.g., autism spectrum disorder, brain injury, cerebral palsy), and English being the primary language spoken at home. During the screening visit, parents were also asked to fill in a demographic questionnaire, from which we used mother’s highest level of education as a proxy for socioeconomic status (SES). We assessed children’s musical experience and calculated a musical experience score (MES) using the same parent questionnaire as Gordon et al.^3^ The descriptive statistics of all the eligible participants (N = 132) including age, SES, and MES is tabulated in Table 1. For details of the screening and eligibility determination please see Supplementary Information.

**Table 1:** Demographics and Assessment Scores of the Study Cohort.

Sample characteristics	N	Mean	SD	SE	Range	Skew	Kurtosis
Age (years)	132	6.53	0.84	0.07	5.07–8.17	0.21	− 1.20
PTONI standard score	124	120.55	19.36	1.74	80–150	− 0.36	− 1.02
Picture vocabulary (scaled score)	132	12.89	2.18	0.19	5–17	− 0.94	1.25
Sentence Imitation (scaled score)	131	12.65	2.12	0.19	8–17	− 0.03	− 0.74
Morphological completion (scaled score)	132	12.86	1.84	0.16	6–17	− 0.63	0.57
Socioeconomic status (SES)	132	7.50	1.00	0.09	5–9	− 0.02	− 0.72
Musical experience (MES)	131	1.09	1.01	0.09	0–4	0.79	0.10

This table summarises the demographic characteristics and scores of the screening measures employed in the study. The picture vocabulary, sentence imitation and morphological completion scores provide age-normed scaled scores for the cohort. A questionnaire was used to assess the musical engagement and calculate a musical experience score, and the highest level of maternal education was used as a proxy for socioeconomic status. The age-normed standard scores of the PTONI are reported for the non-verbal IQ measure. PTONI = Primary Test of Non-Verbal IQ.

### Individual differences analysis

We used assessments of expressive grammar, musical rhythm perception, prosodic perception, and verbal working memory as the behavioural variables for the individual differences analyses. Expressive grammar was measured with the Structured Photographic Expressive Language Test-3 (SPELT-3)^49^ and musical rhythm perception was measured using the children’s version of the BBA (Beat-based Advantage) test^3,50^. The BBA is a computer-based task, in which participants are asked to identify which rhythms are same and which are different from a pair of rhythms presented over speakers. The computer-based Prosody-Matching task, which is a forced choice ABX sentence prosody matching test, was used to probe prosodic perception. In this task participants had to match a low-pass filtered speech sentence to one of two non-filtered sentences (see Methods and Supplementary Information for details). Finally, verbal working memory was assessed with the forward digit span subtest of the Kaufman Assessment Battery for Children second edition (KABC-II^51^).

The descriptive statistics of the outcome measures are summarised in Table 2. From the 132 participants that completed all study visits and assessments, *n* = 103 participants had valid and complete data for all the tasks and assessments. For the computer-based assessments (BBA and Prosody Matching), n = 23 participants (individual test missingness is: n = 11 for BBA, n = 14 for Prosody Matching) did not have useable data for one or more of the tests either due to technical errors, program malfunctions, or participant’s inability to understand or attend to the behavioural task. The path models exclude missing data in a list-wise manner and use only those observations with complete data; the *n* for path models without controlling for IQ is 109, and is 103, when controlling for IQ. Correlation analyses used all available data and excluded missing data in a pair-wise fashion; thus, *n* differs for each pair of variables.

**Table 2:** Descriptive statistics of behavioural outcome measures.

Variable name	N	Mean	SD	SE	Range	Skew	Kurtosis
SPELT-3 standard score	132	113.13	6.66	0.58	90–126	− 0.78	1.06
SPELT-3 raw score	132	46.06	3.80	0.33	35–53	− 0.77	0.22
SPELT-3 complex syntax sub-score	132	0.79	0.13	0.01	0.42–1	− 0.63	− 0.004
BBA d'	121	0.99	0.85	0.08	− 1.20–2.73	0.12	− 0.64
Prosody Matching d'	118	1.79	0.78	0.07	0.13–3.28	− 0.40	− 0.39
KABC-II number recall raw score	132	9.36	2.02	0.18	5–16	0.06	0.37

Age-normed standard scores and raw test scores for the expressive language test (SPELT-3) are shown. The complex syntax sub-score is a non-scaled raw score. The d' dcalculated using signal detection is reported for the BBA and Prosody Matching tests. The non-scaled raw score of the forward digit span subtest of the KABC-II is used. SPELT-3 = Structured Photographic Expressive Language Test-3; BBA = Beat-Based Advantage; KABC = Kaufman Assessment Battery for Children.

### Correlations

**Figure 1:**
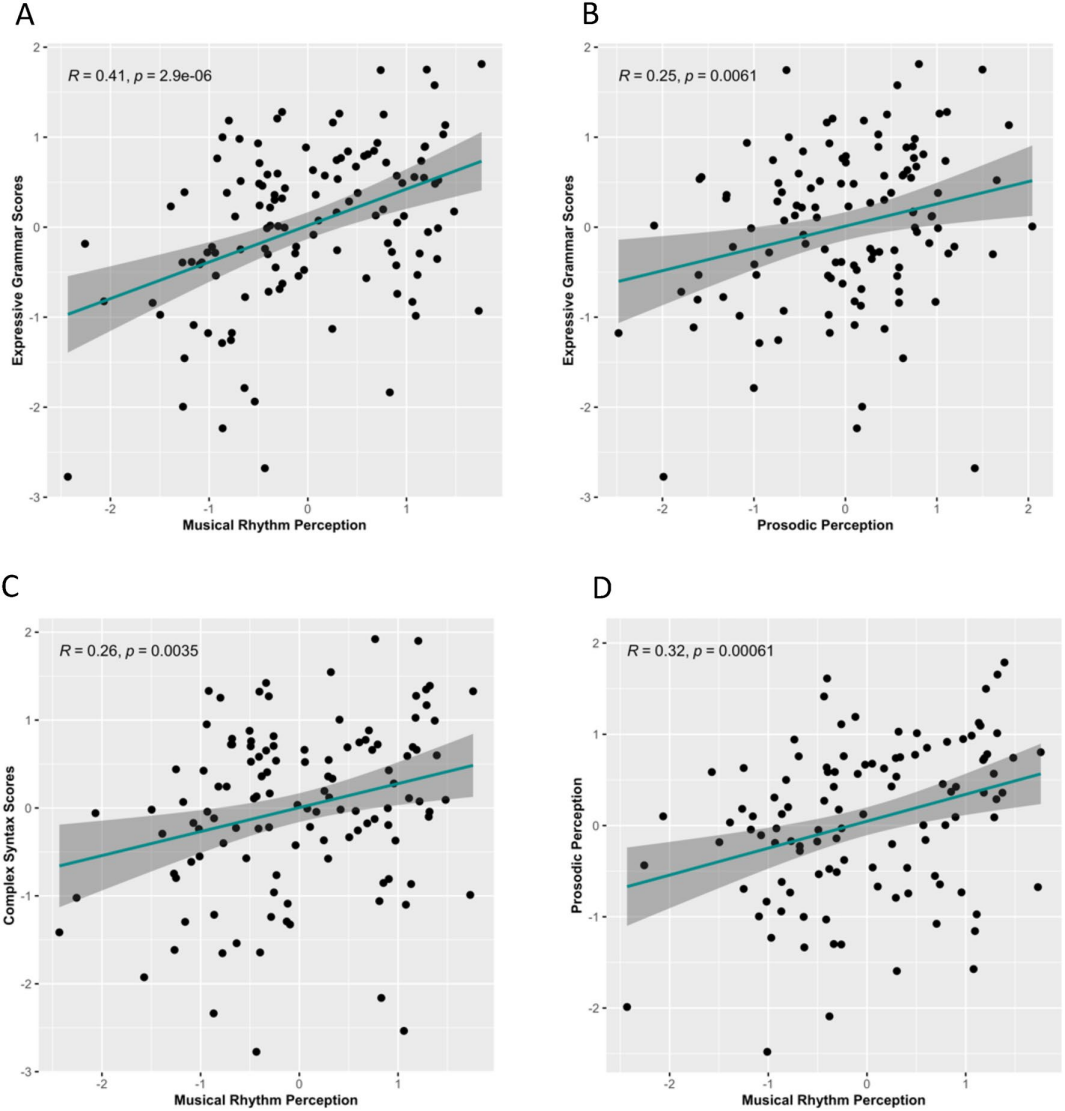
Scatterplots showing the correlations between variables. Scatter plots for correlations between (**A**) expressive grammar (SPELT-3) scores and musical rhythm perception (*n* = 121); (**B**) expressive grammar and prosodic perception (*n* = 118); (**C**) complex syntax sub-scores and musical rhythm perception (*n* = 121); and (**D**) musical rhythm perception and prosody perception (*n* = 109). Age is controlled for in all plots, and Pearson’s r and *p* values are displayed for each correlation.

Correlations between all measures (controlling for age) are displayed in Table 3. For the correlation we first z-scored the raw scores for SPELT-3, BBA, Prosody Matching, KABC-II, MES, and SES, then regressed age from all the variables, and z-scored the residuals obtained from the regression. These z-scored residuals from the age-controlled regression were used for pairwise correlation. For IQ we used z-scored standard scores of the PTONI and used these in the correlation. Since MES and SES did not correlate significantly with prosodic perception, grammar, or musical rhythm perception, they were not included in further analyses. Individual BBA (r = 0.41, *p* < 0.001) and Prosody Matching (r = 0.25, *p* = 0.006) scores were both significantly and positively correlated with expressive grammar (SPELT-3) scores. Prosody Matching scores were also positively correlated with BBA scores (r = 0.32, *p* < 0.001). Further, we also computed a complex syntax sub-score from the SPELT-3 using the item groupings as described in Gordon et al., 2015^17^. Since complex syntactical ability has been demonstrated to be strongly correlated with musical rhythm perception^17^, and processing complex sentences might recruit overlapping neural resources with domain-general rhythm perception abilities, this metric was explored in the associations. In our sample, BBA scores were positively correlated with complex syntax sub-scores (r = 0.26, *p* = 0.003). Interestingly, we also found that the correlation between musical rhythm perception and simple grammar is small and non-significant (see Supplementary Information section ‘Analysis with simple grammar scores’ for details). Scatter plots for the relevant individual correlations are shown in Fig. 1A–D.

**Table 3:** Pearson correlation matrix of all variables used in the correlation and path model analysis.

Variable	1	2	3	4	5	6	7	8
1. SPELT-3 raw score	–							
2. SPELT-3 complex syntax sub-score	0.72**	–						
3. BBA	0.41**	0.26*	–					
4. Prosody Matching	0.25*	0.15	0.32**	–				
5. PTONI standard score	0.27*	0.21*	0.33**	0.24*	–			
6. KABC-II number recall raw score	0.21*	0.10	0.33**	0.27*	0.30**	–		
7. SES	0.08	− 0.01	0.03	0.11	0.10	0.21*	–	
8. MES	0.01	− 0.19*	− 0.06	0.07	− 0.10	0.05	0.18*	–

Except for the PTONI age-normed standard scores (which are the z-scored standard scores), the raw scores of all other variables were z-scored, regressed on age, and the resulting residuals were z-scored once again, and these z-scored, age-regressed residuals were used in the correlation matrix. Values are correlation r values for each pair of variables. Missing values were excluded in a pair-wise manner. SPELT-3 = Structured Photographic Expressive Language Test; BBA = Beat-Based Advantage; PTONI = Primary Measure of Non-Verbal IQ; KABC = Kaufman Assessment Battery for Children; SES = Socioeconomic Status; MES = Musical Experience Score.

*indicates *p* < 0.05, and **indicates *p* < 0.001.

### Mediation analysis

**Figure 2:**
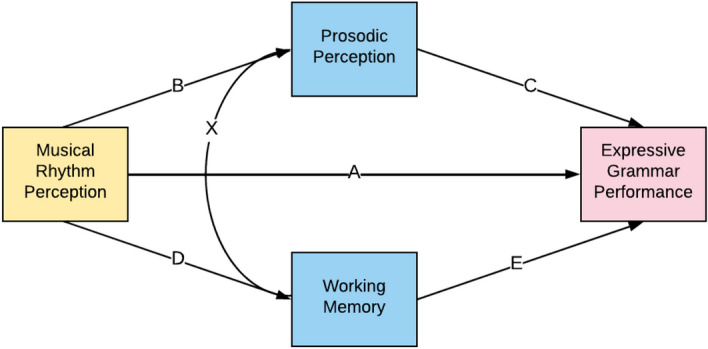
Path model for mediation analysis of musical rhythm perception and expressive grammar performance. This path model examines the relationship between musical rhythm perception (predictor) and grammar performance (outcome), and the possible mediators (working memory capacity and prosody perception) that might play a role in this correlation. Path A is the direct effect from rhythm to grammar, while paths BC and DE constitute the mediation effects through prosodic perception and working memory respectively. Path X depicts the residual correlation between prosodic perception and working memory.

**Figure 3:**
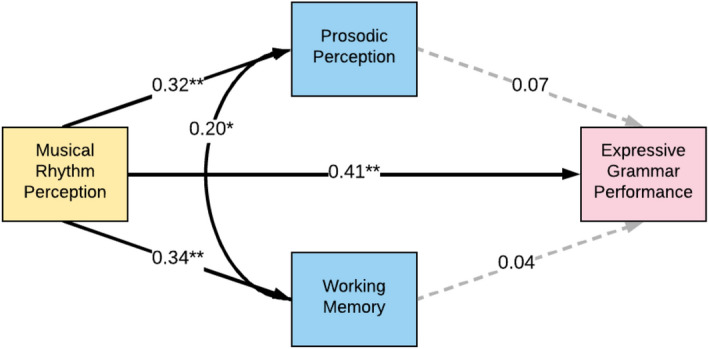
Path analysis model of associations between musical rhythm perception and expressive grammar. The path analysis model shows the associations between musical rhythm perception, expressive grammar task performance, prosodic perception and working memory, while controlling for age (*n* = 109). Z-scored age-regressed residuals of the z-scored raw scores were used for all measures. We found that though the relationship between musical rhythm perception and expressive grammar task performance (path A) is positive and significant, it is not explained by prosodic perception (path BC) or working memory (path DE). The model uses list-wise exclusion for missing values. The β for each pair of variables is indicated on the path. Solid lines indicate a significant relationship. *signifies *p* < 0.05, while **signifies *p* < 0.001.

We used a fully identified path model to investigate the possible mediation of prosodic perception or working memory in the rhythm-grammar correlation, displayed in Fig. 2 (*n* = 109). In this model, we controlled for age by using residualised variables for all measures (i.e., regressing all z-scored raw measure scores on age and computing residualised scores prior to analysis, as described for the simple correlations above). As shown in Fig. 3, the direct path from musical rhythm perception to expressive grammar task performance was significant (Path A; β = 0.41, *p* < 0.001), even when accounting for prosodic perception and working memory. The indirect effects of musical rhythm perception on grammar through prosodic perception (Path BC; β = 0.02) and working memory (Path DE; β = 0.01) were small and non-significant. Thus, musical rhythm perception was correlated significantly with expressive grammar skills, and this correlation was not mediated by either prosodic perception or working memory. The residual correlation between prosodic perception and working memory was also significant (Path X, β = 0.20, *p* = 0.037).

**Figure 4:**
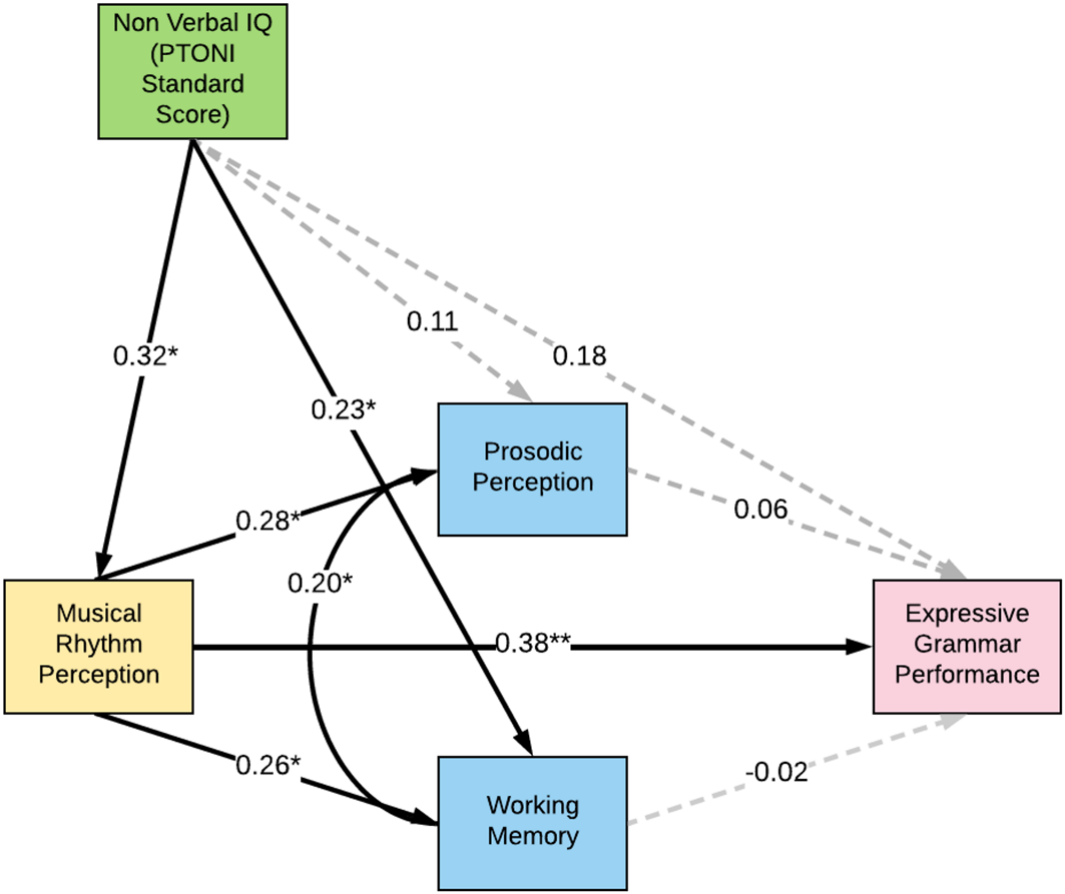
Path analysis model for musical rhythm perception and expressive grammar, controlling for non-verbal IQ. This figure depicts the path model for music rhythm perception and expressive grammar, prosodic perception and working memory as mediators, with IQ as a covariate (*n* = 103). Age was partialled out from all variables (except IQ), by creating z-scored residualised scores of age against the z-scored, raw scores of variables. Z-scored standard PTONI scores were used as the measure of IQ. Missing values were excluded in a list-wise fashion. The β for each pair of variables is indicated on the path. Solid lines indicate a significant relationship.*signifies *p* < 0.05, while **signifies *p* < 0.001.

We conducted the same path analysis by covarying for non-verbal IQ from all measures in the model (*n* = 103). We used the z-scored standard scores from the PTONI as the measure for IQ, while all other variables were the z-scored, age-residualised raw scores. As shown in Fig. 4, although IQ was significantly correlated with musical rhythm perception and working memory, the primary results of the model remain unchanged. That is, the direct path (path A) between musical rhythm perception and expressive grammar remained significant (β = 0.38, *p* < 0.001), while the indirect paths through prosodic perception (path BC) and working memory (path DE) were non-significant. Finally, we also conducted a multiple regression with musical rhythm perception, working memory, and prosodic perception (See Table S1 in Supplementary Information), which also demonstrated that rhythm perception was the only significant predictor of grammar controlling for all other variables, including SES, MES, and nonverbal IQ.

**Figure 5:**
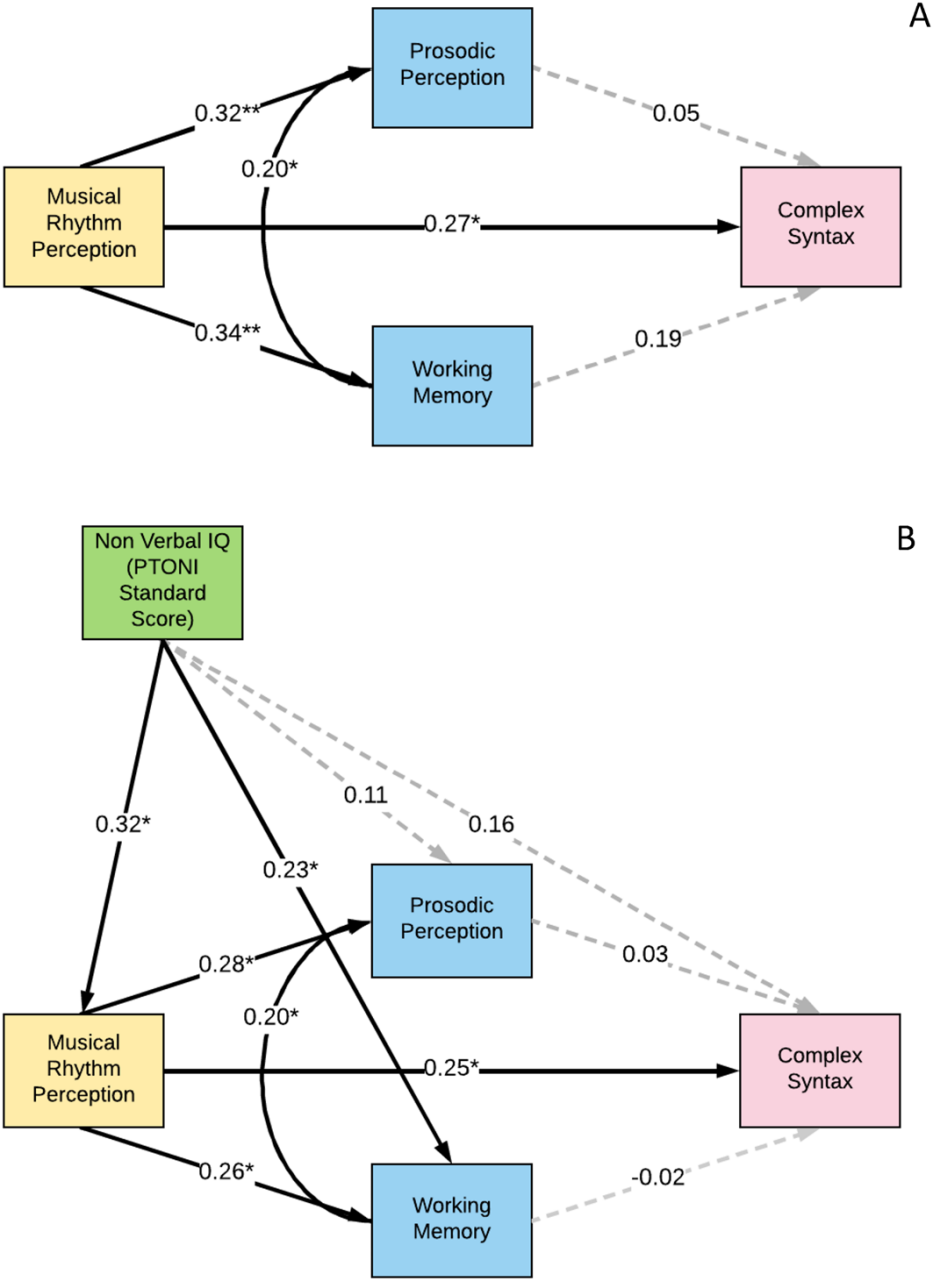
Path analysis model for musical rhythm perception and complex syntax, with and without nonverbal IQ as a covariate. (**A**) Panel A (*n* = 109) depicts the path model for musical rhythm perception and complex syntax, with prosodic perception and working memory as mediators. Residualised scores were created by partialling age from z-scored raw scores of all variables. The age-residualised scores were then z-scored and used in the path model. We find that the relationship between musical rhythm perception and complex syntax task performance is non-significant. Solid lines indicate a significant relationship. *signifies *p* < 0.05, while **signifies *p* < 0.001. (**B**). Panel B shows the path analysis model for musical rhythm perception and complex syntax, controlling for IQ (*n* = 103). Residualised scores were created by partialling age from the z-scores of all variables, except for IQ. For IQ we used z-scored standard PTONI scores. The z-scored, age-regressed residual scores, and z-scored PTONI standard scores were used in the path model. The β for each pair of variables is indicated on the path. Even by controlling for IQ, the relationship between musical rhythm perception and complex syntax remains non-significant. Solid lines indicate a significant relationship. *signifies *p* < 0.05, while **signifies *p* < 0.001.

We performed similar path analyses (controlling for age, as previously described), to investigate the association between musical rhythm perception as the predictor and complex syntax task performance as the outcome, displayed in Fig. 5. We hypothesised that since complex syntax and musical rhythm follow an inherent hierarchy that unfolds over time, processing both complex syntax and rhythm might employ shared processing pathways evident in their behavioural output. Our analysis showed that the direct association, path A between musical rhythm perception and complex syntax was positive and significant (β = 0.27, *p* = 0.007) in our path model (Fig. 5A). Further, as seen in Fig. 5A, the direct association between rhythm perception and complex syntax, remained significant, and the indirect mediating pathways through prosodic perception (path BC, β = 0.02) and working memory (path DE, β = 0.06) were non-significant. Similar results were observed when we included non-verbal IQ as a covariate in the path model (Fig. 5B), showing that complex syntax, much like expressive grammar in the previous path model (Fig. 4), was positively and significantly correlated with performance on the musical rhythm perception task (the BBA) (path A, β = 0.25, *p* = 0.018), and this correlation was not mediated either by prosody perception or working memory, and neither explained by non-verbal IQ. Further, path analyses conducted with simple grammar as an additional covariate, demonstrated that there is a unique correlation between musical rhythm perception and complex grammar (See supplemental information Fig. S5).

### Power analysis

Post-hoc power calculations for testing the mediation effect (e.g., through prosody or working memory) were conducted using the powerMediation package in R^52^. Achieving 80% power for an indirect effect of β = 0.10 (alpha = 0.05; two-tailed test; path from rhythm to mediation β = 0.32, path from mediator to grammar β = 0.32) requires N = 72 participants, less than our achieved N = 109. Based on the actual indirect effects described in Fig. 3, which were quite small (β = 0.022 for prosody, β = 0.014 for working memory), we would need N = 140 or N = 282 individuals, respectively, to detect a significant indirect path. Thus, our study was powered to detect an indirect effect of small-to-medium size, but not the very small indirect effects observed here.

## Discussion

In alignment with prior work^3,4,13–15^, here we demonstrated a positive correlation between musical rhythm perception and expressive grammar skills in a sizeable sample of 132 school-aged (5–7-year-old) children. We also found that prosodic perception is positively correlated with musical rhythm perception (r = 0.32, *p* < 0.001), and with expressive grammar (r = 0.25, *p* = 0.006). The importance of prosodic perception in the context of language has been elaborated in studies which showed that prosodic cues play an important role in early language acquisition^53^ and account for variance in literacy skills in children^54^. Imposing metrical regularities in spoken sentences also affects adult listeners’ neural processing of grammatical structure^30^, indicating that prosodic sensitivity continues to be important for language processing later in the lifespan^20,55^. Previous studies have explored the correlation between prosodic sensitivity and reading and literacy skills^56^ as well as prosodic sensitivity and musical rhythm skill^35^, showing that weak performance on prosodic tasks tends to co-occur with weak reading comprehension and musical rhythm perception skills. Based on these observation, we explored the possibility that prosodic perception would be an important factor mediating the associations between musical rhythm and grammatical skills.

To study the nature of this relationship, we used a path model that factored in prosodic perception as a mediator, and tested working memory as an additional potential mediator, since working memory also tends to play a role in performance on musical and grammar tasks^40,43,57^. Contrary to our predictions about these potential mediators, the associations between musical rhythm and grammar scores remained significant and were not accounted for by prosodic perception ability, nor by working memory, evidenced by the results of the path model. Importantly, covarying non-verbal IQ and age did not impact these associations, thus indicating that the dynamics of the relationship between musical rhythm and spoken grammar skills are not driven by prosodic perception, verbal working memory, general cognition, or non-specific developmental effects. These findings replicate and extend other recent work that has shown a relationship between rhythm-related skills and spoken grammar task performance, above and beyond general cognitive effects or working memory^2–4^.

Furthermore, in our study, the path model with complex syntax sub-scores demonstrated similar results as for overall grammatical skill as the outcome: there was a significant direct effect of musical rhythm perception on complex syntax, and this relationship was not mediated by prosodic perception nor working memory. In line with previous findings of musical rhythm variables predicting children’s individual differences in complex syntax skill^17,58^ and adults’ years of musical experience predicting attainment of long-distance syntactic dependencies in an artificial language learning paradigm^59^, we thus found converging evidence for an association between mastery of complex multiclausal syntactic structure and musicality variables.

Overall, our cross-sectional results should not be interpreted as a causal role of musical rhythm skill on children’s grammatical development. While the present study upholds previous observations that rhythm and grammar performance are indeed correlated, we did not delve into the neural, biological, or genetic mechanisms that are responsible for this association. Recent twin studies, for example, have revealed that shared genetic influences account for much of the association between verbal ability and measures of musical aptitude and music engagement^60,61^. Such findings are more consistent with a model in which musical and language traits are influenced by a similar set of genetic (and environmental) factors, rather than causal associations of music on language development. Moving forward, other study designs will be necessary to unpack any potential longitudinal or (bi)directional associations between rhythm and grammatical skills. Further, while we did not observe mediation between musical rhythm perception and grammar via working memory, it is possible that we might have observed this mediation had we used a working memory related task that engaged cognitive manipulation as well. We used the forward digit span as a measure of working memory, a task which relies primarily on the storage capacity aspect. Working memory consists of both—the ability to store information and the ability to manipulate it, and using a more involved task like the backward digit span, in addition to the forward digit span, might have provided a more nuanced point of view.

A particular limitation is that in our application of the structural model to our data, due to not having multiple measures of each construct in the full sample, we could not construct latent variables for our measures. Inclusion of latent variables would have reduced the measurement error present in the study measures and allowed us to create variables that capture individual differences with reduced error margins. Future studies should include multiple correlated measures for each construct (i.e. rhythm production, perception, and imitation for a “rhythm” construct^62^) while also maintaining a large enough sample size to warrant path modelling and other types of structural equation models. Another caveat is that our study only included monolingual speakers of American English. Studies looking at musicality and language skills in bilingual participants^2,63,64^ suggest that a fruitful strategy for future work would be to employ more inclusive inclusion criteria, with bilingualism coded as a covariate, as successfully demonstrated in Swaminathan and Schellenberg^4^.

It is also important to keep in mind that here we quantified prosodic perception with a Prosody Matching task (employed through discrimination of filtered sentences) that focused on sensitivity to prosodic cues signifying syntactic structure, i.e., phrasal lengthening to indicate pauses or intonational markings that signify when a question is being asked. This assessment thus may not capture other rhythmic aspects of speech (i.e., lexical stress^65,66^), but rather capture prosodically-directed sentence-level syntactic processing of the phrase constituents. Future incorporation of speech-rhythm (e.g., tasks concerning identification of stressed syllables, or repetition of nonsense syllable rhythms) will likely help disentangle the effects of prosodic perception on the associations between rhythm and grammar task performance. Similarly, for the outcome measure, we have used expressive grammar abilities, complementing prior research that has shown similar results with receptive grammar tasks^2,4^. Grammatical syntactic processing is a complex, multi-layered neurobiological function, and studying its various facets will help specify the nature of rhythmic skills and how they might impact other individual differences in morpho-syntactic processing/skills.

From the literature and from our study we observe that musical rhythm sensitivity has significant implications for development of grammatical skills, such that children who are more sensitive to musical rhythm seem to have an advantage during language learning, though the processes underlying this relationship are not well-understood. Children’s early-emerging sensitivity to speech prosody is leveraged by infants during language and especially syntactical learning^28,67^. The association between rhythm perception and language becomes more directly measurable by pre-school age; Politimou et al.^1^ demonstrated that rhythm perception and production skills predicted sentence imitation task (which taps into both receptive and expressive grammar and syntax), converging with findings by Woodruff Carr et al.,^68^ that rhythm discrimination and beat synchronisation predict sentence imitation. There is a relatively large body of studies linking musical rhythm to literacy skills; for example rhythmic sequence reproduction is positively associated with children’s reading and spelling performance in school in school-aged children^69^. Taken together, these observations suggest that (a) early language and music development might be entangled, (b) language development to some extent utilises rhythmic perception, and (c) the domain-specificity and nuanced processing of these stimuli develops over time^70^.

Both musical rhythm and linguistic syntax are ordered in a temporally progressing hierarchy^71–74^. Considering these structural parallels, there could be shared mechanisms at play that are responsible for processing both music and grammar^75,76^. One cognitive pathway that might feature in the relationship between musical rhythm and grammar processing is hierarchical processing, which helps to break down a complex, stratified system into its individual components^77^. With some similarities to rhythm, the grammatical structure of language, especially complex syntax, follows a hierarchical structure that unfolds over time^73,77,78^. Our finding of a direct relationship between children’s rhythm discrimination and complex syntax skills suggests a possible role of hierarchical processing across domains. There is also functional support for this possibility from a recent meta-analysis which demonstrated some overlap between brain regions involved in processing musical rhythm and syntactic structure in language (i.e., inferior frontal gyrus, supplementary motor area, and bilateral insula), with the common cognitive mechanism attributed to hierarchical processing^79^. Evolutionary accounts of a common origin of language and music have pointed to a preference for tree-like hierarchical structures in early humans’ communication traits that may have subsequently given way to rhythm and language in modern language^32,77,80^.

The underlying biology of the cross-trait relationship between rhythmic perception and grammatical skills, especially in light of proposed shared evolution, could be explained by genetics—both through shared genetic architecture^60,81^ between human rhythm and grammatical traits, and through evolution of hierarchical processing as a common mechanism to process syntax in both music and grammar^82^. Developmental observations in musicality and language skill further support the case for common genetic influences on these traits, perhaps that are ontogenetic. Although as adults we might consider musical rhythm and speech highly dissimilar^83^, evidence collected from studies with infants looking at language discrimination, speech tracking and rhythmicity^84,85^, melodic complexity of crying and language acquisition^86–88^, and familiarity with musical rhythms and words^89^ might indicate that development of rhythm and grammatical skills is concomitant rather than sequential.

In conclusion, our study in school-aged children adds to the growing literature in music cognition that demonstrates the association between individual differences in musical rhythm perception and expressive grammatical skill. Our results demonstrated that the ability to parse complex syntax is also correlated with musical rhythm perception, indicating that there might be a common mode of processing syntactical hierarchy in both rhythm and grammar. This study replicates the finding that there is a positive correlation between rhythm and grammar performance, but interestingly this relationship is independent of working memory and IQ, is not simply driven by sensitivity to prosody but rather is truly a cross-domain phenomenon. These results open the door to future investigations of underlying common biological and genetic mechanisms that drive rhythm-grammar links with a focus on the dynamic underlying causes of this relationship.

### Methods

The study protocol was reviewed and approved by the Vanderbilt University Institutional Review Board (IRB #120,062). All experiments were performed in accordance with Vanderbilt IRB regulations and all relevant ethics guidelines from the Belmont Report. For the study, all participants, and their parents gave verbal assent and written informed consent respectively, in accordance with established protocols.

### Participant recruitment

Children (N = 150) between the ages of 5–7 were recruited from several sources in the Middle Tennessee community such as libraries, museums, research mailing lists through Vanderbilt University, and schools, and were screened for the study. Children were invited for one in-depth, on-campus screening visit, and those who were eligible were invited back for one or two additional study visits on-campus. Note that all the children were between the ages of 5 and 7 when their language was characterised, and they were enrolled during the screening visit. However, one child turned 8 by the time the additional study visits were completed. This is reflected in the age range reported for the expressive language outcome measure (SPELT-3). Study data were collected and managed using REDCap electronic data capture tools hosted at Vanderbilt University^90,91^. Families were compensated with a gift card and small toy per visit. Details of screening measures and language characterisations used to determine eligibility for the study are detailed below; N = 132 were included in the final dataset. Note that data from the same cohort, including the language screening characterisations, non-verbal IQ and the expressive grammar (SPELT-3) variables, are also utilised as the sample with typical language development in another study focused on motor timing in developmental language disorder^92^.

### Screening and language characterisation

#### Hearing screening

Using headphones, each child listened to a series of 20 dB pure-tones presented 3 times at 1000, 2000 and 4000 Hz in each ear respectively (18 tones in total; 9 in each ear; 3 at each frequency). To pass, participants needed to correctly identify two out of three presentations at each frequency, in both ears. If a child failed a hearing screening due to allergies/infections, they were invited back for a second screening, which if they passed, allowed them to continue in the study.

#### Nonverbal IQ

To rule out cognitive disabilities and obtain a covariate of non-verbal cognition, the PTONI (Primary Test of Non-Verbal IQ)^93^ was administered to assess age-normed nonverbal IQ; details are provided in the Supplementary Information.

#### Language characterisation

The TOLD-P:4 (Test of Language Development—Primary, 4th edition; Newcomer & Hammill, 2008)^94^ and the TEGI (Test of Early Grammatical Impairment; Rice & Wexler, 2001)^95^ were used to assess language eligibility and rule out language disorders. To assess typical language development, we used three subtests from the TOLD-P:4, namely Sentence Imitation (SI), Picture Vocabulary (PV) and Morphological Completion (MC). Since these subtests assess expressive and receptive syntax, receptive vocabulary, and expressive morphology respectively, they effectively capture language impairments, if present. We also used the phonological probe from the TEGI to rule out speech and/or phonological impairments that may interfere with accurate assessment of morphosyntax.

Children who failed the phonological probe of the TEGI (raw score of 12 or below) were excluded from study participation. Children with signs of language impairment as judged by SLP clinicians were not eligible to continue and were referred to another study for children with language impairment. Evidence of potential language impairment was indicated by below average scaled scores on any one of the specific TOLD subtests (scaled score < 8 for either SI, PV, or MC). One participant scored below criterion on the SI subtest for the TOLD P-4 due to non-compliance, and four children scored below average on one out of three subtests. However, their average performance on the PTONI, SLP expert opinion based on holistic review of other tests, and observations during the assessments verified normal language development, which allowed them to be included in the study. All standardised language testing and decisions to include or exclude participants based on scores were overseen by Speech-Language Pathologist research staff. Language characterisation was performed for eligibility, whereas expressive grammar, the phenotype of interest for the individual differences analysis, was measured with the SPELT-3 (described below).

It is important to note that our inclusion criteria focussed on *typical language development*, and hence the TOLD-P:4 and the phonological probe from the TEGI, were used for language characterisation. This phonological probe tests the child’s ability to produce certain phonemes and is, as such, a test of developmental impairments of articulation, and not of phonological awareness, and cannot detect presence of reading impairments. There is therefore, a possibility that children with undetected reading difficulties were included in our sample, even though their spoken language was found to be typically developing. For more details, see Supplementary Information.

### Behavioural assessments for individual differences analyses

#### Grammar

Expressive grammar was measured with the Structured Photographic Expressive Language Test-3 (SPELT-3)^49^. Participants were shown a series of pictures and asked questions that elicit responses requiring specific grammatical construction (e.g., relative clauses, possessive pronouns). The z-scored raw scores from this assessment were used as the outcome measure of expressive grammar skills for the phenotypic correlations, while controlling for age. We also generated complex syntax subscores from the SPELT-3 using the 12 items designated to have complex syntax form^17^.

#### Rhythm assessments

##### Musical rhythm perception (BBA)

Musical rhythm perception was measured using and the children’s version of BBA (Beat-based Advantage) test^3,50^, which has been adapted from the original adult version of the task implemented by Grahn and Brett^96^, and use auditory stimuli originally developed by Povel and Essens^97^. The children’s BBA is a computer-based test developed and presented via E-Prime v2.0 Professional (Psychology Software Tools, Inc.) on a laptop. The sounds were presented over external speakers connected to the laptop and calibrated to 70 dB. The BBA was presented to children as the “Drummer” game (see Supplementary Information for visuals and details). During the task, participants were asked to listen to three standard rhythms, played sequentially. The first two rhythms were identical and played by a character called Randy Drummer, while the third and final rhythm could be the same as the first two (thus played by Sandy Same who copies Randy Drummer), or different (played by Doggy Different). Children were asked to determine whether the third rhythm was played by Sandy Same or by Doggy Different. This task employed simple and complex rhythms (half the trials each). The same stimulus corpus and visual representation was used here as in Gordon et al.^3^ such that rhythms were 25% faster than in the other work in adults, due to young children’s faster internal tempo^98^.

The BBA had 4 practice trials (2 simple and 2 complex—each with a same/different variation) followed by 28 test trials, divided into 14 simple and 14 complex rhythms, with each rhythm type further subdivided into 7 “same” and 7 “different” trials. The rhythms heard in the practice trials were excluded from the test trials. Correct/incorrect feedback was provided only in the practice stage. For analyses, we calculated the d’ separately for simple and complex rhythms, with children generally performing better on trials with simple rhythms (higher d’ values) than with complex rhythms. The simple and complex d’ were then averaged for an overall BBA d’ score. This BBA raw score was further z-scored, controlled for age, and used as the measure of musical rhythm perception in our analyses.

##### Prosodic perception

Prosodic perception was measured with a Prosody-Matching task (adapted from Soman^99^), which tests sensitivity to intonational fluctuations in speech. The task was presented through the “Astronaut” game, via E-Prime using the same computer and speaker setup as for the BBA. The task was a forced-choice ABX discrimination task that required children to match the prosody of a low-pass speech filtered sentence to one of two non-filtered, normal speech sentences (see Supplementary Information for visuals and details of the task). The unfiltered sentences were spoken by either the red astronaut or the blue astronaut in the visual, while the low-pass filtered speech was attributed to their green alien friend. Children were told that the astronauts and their green alien friend were playing a copycat game, and that the alien was trying to copy what one of the astronauts was saying, but it's hard to hear in outer space, and it was the child’s job to determine which astronaut the alien was mimicking. The task had a total of 24 trials (12 trials mimicking the red astronaut, and 12 trials the blue) which were presented to the participants in random order. Similar signal detection theory analysis was used to calculate the d’, which was then z-scored, controlled for age, and the residuals were further z-scored. These z-scored, age-controlled residuals were used as a measure of prosodic perception. For experimental details of the BBA and Prosody Matching task, please see the Supplementary Information.

#### Verbal working memory

The forward digit span subtest of the KABC-II (Kaufman Assessment Battery for Children, second edition)^51^, was used to measure verbal working memory, given that working memory has been positively correlated with rhythm perception and synchronisation and with grammatical task performance in earlier work^43,45^. During the task, children heard a recording through the computer speakers of a number-series that they were asked to repeat back to the examiner verbatim. The raw scores from the test were z-scored, controlled for age, and the resulting residuals were z-scored and used for analysis.

### Analysis plan

All data were analysed using R software environment^100^, the tidyverse R package^101^, and the lavaan package^102^. Using lavaan, a structured equation model (SEM)/path model was used to examine the possible mediation of the music rhythm perception—grammatical skill relationship through prosodic perception or working memory, with missing values excluded in a list-wise manner. Prosodic perception and working memory served as possible mediators, with IQ and age as covariates. As shown in Fig. 2, we evaluated two possible mechanisms of mediation—one through prosodic perception (path BC), and the other through working memory (path DE). This path model was fully identified, hence model fit indices were not considered.

For both the path model and correlation analyses, we used raw scores for all our measures (SPELT-3, KABC-II, Prosody Matching, BBA), except for non-verbal IQ, where we used the age-normed PTONI scores. For the behavioural measures which used raw scores (i.e., SPELT-3, KABC-II, Prosody Matching, and BBA), we first z-scored, i.e., standardised the raw scores. Then age was partialled from the behavioural measures by generating residuals from the correlation between the z-score of age and the z-score of the behavioural measures. Finally, these residuals were z-scored again, and these residual z-scores were used as the final input values of the behavioural measures in the analyses. For the PTONI standard scores, we z-scored the available PTONI scores, and then used these z-scores in our analyses. Though the SPELT-3 is a standardised test, with age normed scores available, since we used the SPELT-3 scores as an outcome measure of expressive grammatical ability for individual participants as a representative of the population, rather than as a measure of ranking/standing within the general age group of the participants, we used raw scores which were then controlled for age (as described above) in our analysis.

## Supplementary Information

Supplementary Information.

## Data Availability

Data analysis code and de-identified variables reported in this manuscript are made available on Open Science Framework at 10.17605/OSF.IO/KG6JX.
