# Supplementary material for: Exploring individual differences in musical rhythm and grammar skills in school-aged children with typically developing language
Source: Sci Rep. 2023 Feb 7;13:2201. doi: 10.1038/s41598-022-21902-0 (PMC9905575; doi:10.1038/s41598-022-21902-0)
Supplement: Supplementary file 1 — Supplementary Information. [file 41598_2022_21902_MOESM1_ESM.docx]

**Supplementary Information**

***Exploring Individual Differences in Musical Rhythm and Grammar Skills in School-aged Children with Typically Developing Language***

Rachana Nitin, Daniel E. Gustavson, Allison S. Aaron, Olivia A. Boorom, Catherine T. Bush, Natalie Wiens, Chloe Vaughan, Valentina Persici, Scott D. Blain, Uma Soman, David Z. Hambrick, Stephen M. Camarata, J. Devin McAuley, Reyna L. Gordon.

**Participant Screening:**

From the 150 participants that were screened, 2 participants were considered ineligible due to repeated failed hearing screenings, while 7 were “lost-to-follow-up” and did not return for further testing; thus 141 participants completed the behavioural assessments of the study. A further 9 participants were excluded from analysis, due to the following reasons: speakers of dialects other than Standard American English (n=3), 2^nd^ -language immersion program at school (n=1), difficulties in understanding and/or completing the tasks (n=1), failure to meet TD language criteria on assessments (n=3), and technical errors during administration of computer assessments (n=1). Thus N = 132 qualified for the full study.


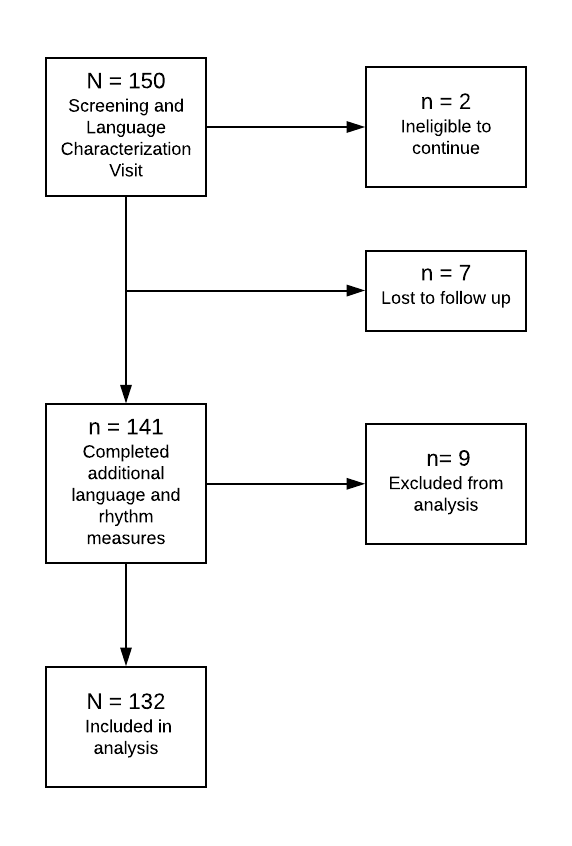


**Figure S1.**  **Study enrolment pipeline for participants**. This figure encapsulates the review pipeline for participant inclusion, from screening to final analysis. All decisions to exclude participants based on eligibility were made by SLPs after review of the participants’ screening measurements and assessments.

**Nonverbal IQ:**The Primary Test of Non-Verbal IQ (PTONI)^1^ was used to assess age-normed performance IQ. In the PTONI participants were asked to determine which picture does not belong in a series of pictures. Participants needed a standardized score >= 78, which is indicative of absence of intellectual disabilities, to qualify for the study. Of the 132 total eligible participants, *n*=8 participants had invalid administration for the PTONI (due to incorrectly established basal scores, child shyness, or inattention); however, study eligibility was established through a combination of clinical judgments and above criterion performance on the pertinent subtests of the TOLD P-4 (see Language Characterization in the Methods section for details). These scores rule out global intellectual disability, even when a PTONI score was not available.

**Test of Word Reading Efficiency:**

Our inclusion criteria for this study were focussed on screening for typical language development. In addition to the phonological probe from the TEGI which was used to screen for presence of articulation impairments, all participants in our sample were also administered the Test of Word Reading Efficiency -2 (TOWRE-2), as part of a separate study. Of the 132 participants that met eligibility criteria for this study, n=45 were below the age of 6 and could not be administered the TOWRE-2; n=87 were above 6 and took the TOWRE-2. Of the 87, n=81 have valid TOWRE scores, with only one child having a standard score below 80 (score of 76), and n=6 did not have valid TOWRE scores since they could not complete the practice items of the test. Thus, overall, the sample is characterised by typical reading skills.

**Rhythm Phenotypes:**

**Beat Based Advantage (BBA) Task**: The BBA^2,3^ was presented to participants as the “Drummer” game where there are three parties involved – Randy-Drummer, Sandy-Same and Doggy-Different^2^. The first two identical rhythms are played by Randy-Drummer, and the third rhythm is either played by Sandy-Same, who copies Randy and plays the same rhythm, or by Doggy-Different, who plays a deviant rhythm. It was the participants’ job to determine who was playing the third and final rhythm, using a clicker.


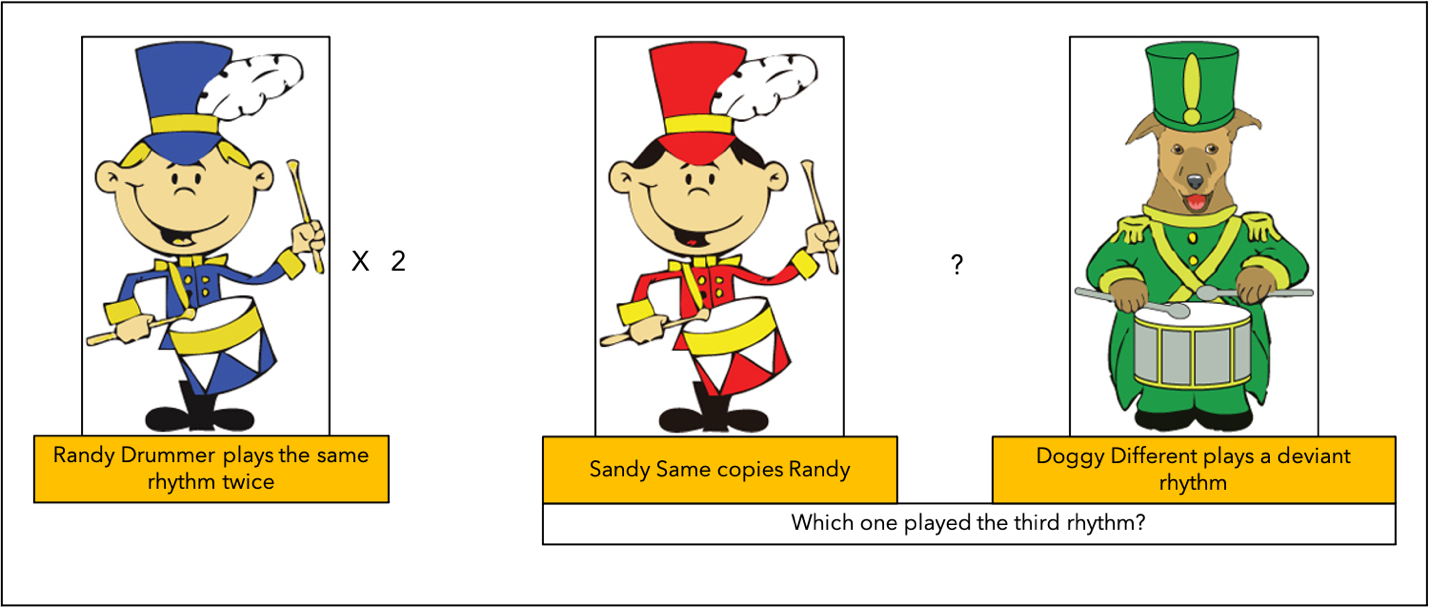


**Figure S2. Illustration of the BBA task and the visual stimuli shown to participants when listening to the rhythms**. For each trial, children heard a standard rhythm repeated 2 times, followed by a third rhythm which would either be the same as the first, or different. Participants were asked to choose between “Sandy Same” and “Doggy Different” with a clicker to indicate if they thought the third rhythm was the same as the first two or different.

Note: The BBA paradigm was used in Gordon et al. 2015^2^, and this figure was designed by Dr. R.L. Gordon and Dr. E. A. Wieland for the use of the BBA paradigm with children. The drummer illustration was loosely adapted from an online template (<https://www.shutterstock.com/image-vector/happy-drummer-drumming-65248204>), while the dog in the illustration is an original creation.

**Prosody Matching Task:** The Prosody Matching task^4^ was a forced-choice ABX discrimination task that involved listening to two normal-speech sentences, and a third sentence which is subjected to a low-pass 400Hz filter. This filtered sentence mimicked the prosody of one of the two normal-speech sentences. The children were presented with the first unfiltered sentence (Stimulus A), and then a second sentence (Stimulus B). The third phrase the participants heard (Target X), is the low pass, 400Hz filtered version of either Stimulus A or B. Thus, Target X is mimicking the prosody of either Stimulus A or B, and the children were asked to determine which prosody ‘X’ was mimicking.

The prosody matching task was presented through the “Astronaut” game. The participants were told that there are two astronauts – a red astronaut and a blue astronaut, and they have a “green alien friend”. The green alien and his astronaut friends are playing a copycat game where the alien is trying to mimic what one of the two astronauts is saying, but because they are in outer space the alien cannot be heard clearly. The children were presented with the first unfiltered sentence (Stimulus A) spoken by one of the astronauts – either red or blue (the order is switched for successive participants), and then a second sentence spoken by the other astronaut (see Figure S3).

Stimulus A is always a declarative sentence, and the second utterance (Stimulus B) is a modified version of Stimulus A – it is either a shorter version of the first declarative sentence, has an alternative pausing schema, or adds interrogative intonation to the sentence. Both Stimulus A and B are presented in normal, unaltered speech. An example of the stimuli is provided below:

Stimulus A - Declarative sentence: “The boy is pouring juice in the glass.”
 Stimulus B - One of the following modified conditions:

1. Short version: “The boy is pouring juice.”
2. Alternative pausing: The boy is pouring // juice in the glass.”
3. Interrogative intonation: “The boy is pouring juice in the glass?”

The third phrase the participants heard (Target X), is the voice of the green alien, and is the 400 Hz filtered version of either Stimulus A or B. Thus, the green alien (Target X) is mimicking the prosody of either the red astronaut or the blue astronaut, and the children were asked to determine which astronaut the alien was copying. A total of 24 such trials (12 trials mimicking Stimulus A and 12 trials Stimulus B) were presented to the participants in random order.


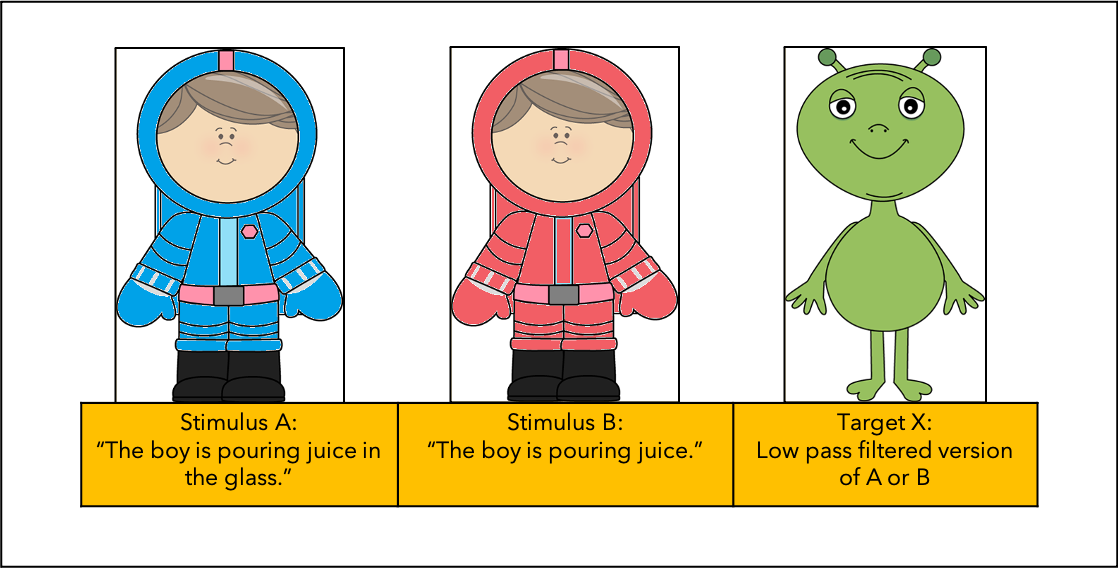


**Figure S3**. **Illustration of the prosody matching task to test speech rhythm sensitivity, and the visual stimuli used in the “Astronaut game”.** For each trial, two normal-speech stimuli A and B (astronauts) were presented, followed by one of those two sentences low-pass filtered through 400 Hz (Target X). Participants were asked to identify which astronaut the alien was imitating.

Note: The illustrations for the Prosody Matching task were designed by Dr. S.D. Blain, N. Wiens, and C. Vaughan, and loosely adapted from an online template. (<https://www.mycutegraphics.com/graphics/space/alien-and-astronaut.html>).

**Analysis with simple grammar scores:**

We included analysis with simple grammar scores to demonstrate the special relationship between musical rhythm perception and complex grammar, as compared to the relationship between musical rhythm perception and simple grammar. For simple grammar we used the verbal morphology, inflectional morphology, nominal morphology and aspect SPELT-3 subscores which were calculated as described in the Gordon et al., 2015^5^ paper. We first z-scored the raw scores for each of these subscores, then averaged across the z-scores to create a single score and then z-scored this average score to generate a simple grammar composite score. Age was then partialled from this composite simple grammar z-score, and the residualised scores were z-scored once again and used in the analysis. For more details pertaining to controlling age, refer to the Analysis Plan section in the Methods.

Correlations between simple grammar scores and musical rhythm perception and prosodic perception are visualised using scatter plots shown in Figure S4 A-B. Individual BBA (r = 0.12, p = 0.18) and Prosody Matching scores (r = 0.11, p = 0.23) were not correlated with simple grammar scores. Additionally, we also conducted a path analysis between musical rhythm perception and complex grammar, with prosodic perception and working memory as mediators and nonverbal IQ ­*and* simple grammar as covariates (Figure S5). Our results show that the direct path from musical rhythm perception to complex syntax task performance was significant (Path A; β = 0.24, p = 0.02), even when accounting for prosodic perception and working memory. The indirect effects of musical rhythm perception on complex syntax through prosodic perception (Path BC; β = 0.008) and working memory (Path DE; β = -0.003) were small and non-significant. The relationship between musical rhythm perception and complex syntax was also not explained by non-verbal IQ, ­*nor* by simple grammar task performance.

B

A


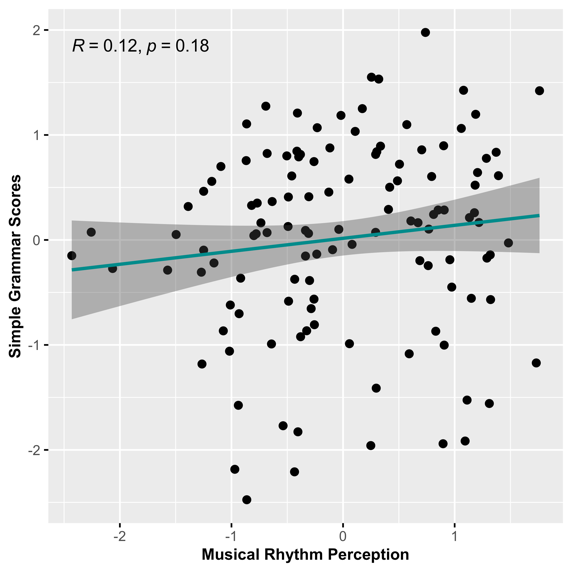

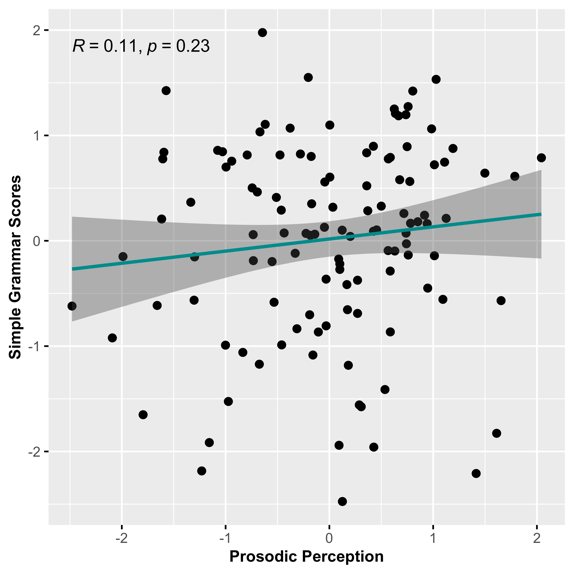


**Figure S4. (A)** Scatterplot showing the correlation between simple grammar scores and musical rhythm perception (*n* = 121); **(B)** Scatterplot for correlation between simple grammar and prosodic perception (*n* = 118). Age is controlled for in all plots, and Pearson’s R and p-values are displayed for each correlation.

**
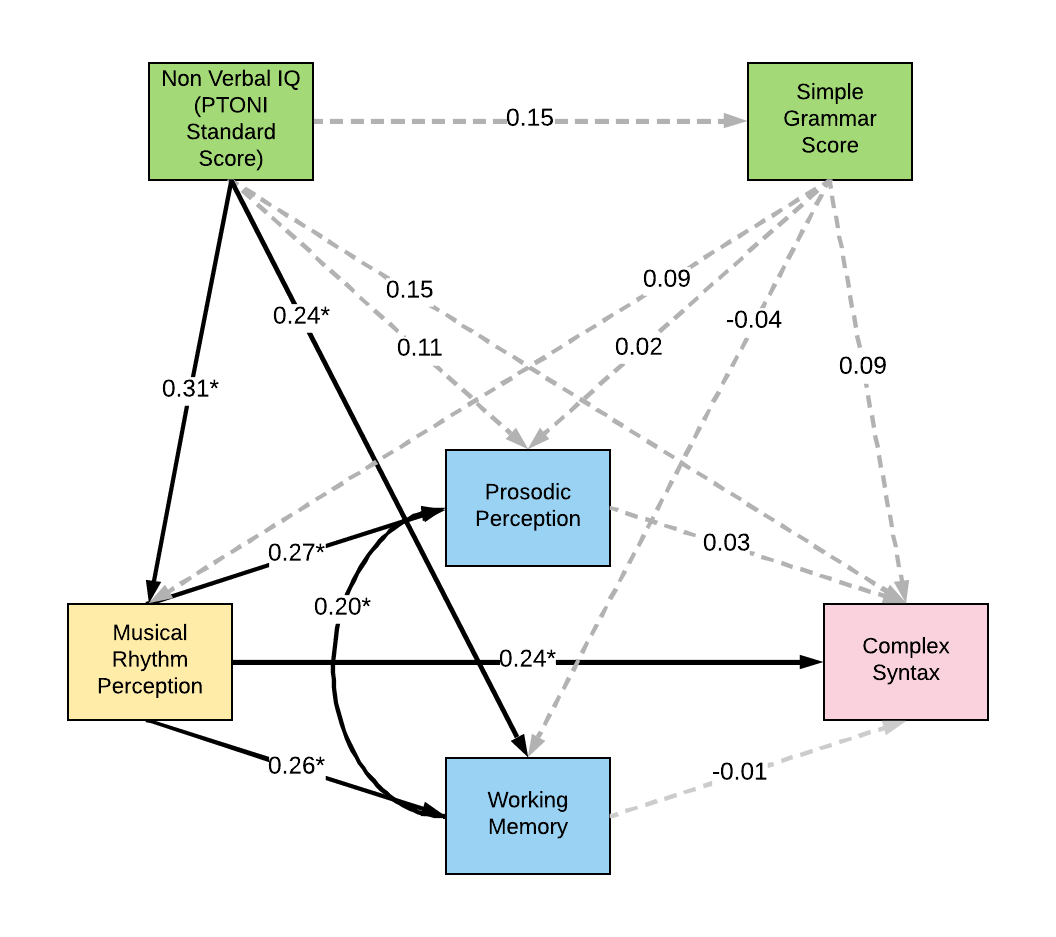
**

**Figure S5**. **Path analysis model for musical rhythm perception and complex syntax, controlling for IQ and simple grammar (*n* = 103).** Residualised scores were created by partialling age from the z-scores of all variables, except for IQ. For IQ we used z-scored standard PTONI scores. The z scored, age-regressed residual scores, and z-scored PTONI standard scores were used in the path model. The β for each pair of variables is indicated on the path. Even by controlling for IQ and simple grammar, the relationship between musical rhythm perception and complex syntax remains non-significant. Solid lines indicate a significant relationship. * signifies p < 0.05, while ** signifies p < 0.001.

**Multiple Regression:** As an exploratory analysis, we ran a multiple regression predicting grammar as a function of musical rhythm perception, prosodic perception, working memory, nonverbal IQ, SES and MES. Raw scores were used for KABC-II, SES, and MES, d’ scores were used for BBA and Prosody Matching tests. All scores were first z-scored, age was partialled from these scores, and the residuals were further z-scored. For the PTONI, the standard scores were simply z-scored, and these z-scores were used in the analysis. For the other measures, the z-scored, residualised scores were used in the regression. For more details pertaining to score calculation and controlling age, refer to the Analysis Plan section in the Methods.

| **Variable** | **β** | **SE** | **p** |
| --- | --- | --- | --- |
| 1. Musical rhythm perception (BBA d’) | 0.40 | 0.10 | 0.00 |
| 2. Prosodic perception (d’) | 0.05 | 0.11 | 0.63 |
| 3. Working memory (KABC-II) | -0.05 | 0.11 | 0.64 |
| 4. Nonverbal IQ (PTONI Standard score) | 0.19 | 0.10 | 0.06 |
| 5. Socioeconomic status (SES) | 0.08 | 0.10 | 0.39 |
| 6. Musical Experience Score (MES) | 0.07 | 0. | 0.50 |

**Table S1. Results (β, standard error, and p value) of the multiple regression for each of the variables predicting grammatical ability**. BBA = Beat-Based Advantage; PTONI = Primary Measure of Non-Verbal IQ; KABC = Kaufman Assessment Battery for Children; SE = Standard Error.

**Bibliography**

1. Ehrler, D. & McGhee, R. PTONI: Primary test of nonverbal intelligence. (2008).

2. Gordon, R. L. *et al.* Musical rhythm discrimination explains individual differences in grammar skills in children. *Dev. Sci.* **18**, 635–644 (2015).

3. Wieland, E. A., McAuley, J. D., Dilley, L. C. & Chang, S.-E. Evidence for a rhythm perception deficit in children who stutter. *Brain Lang.* **144**, 26–34 (2015).

4. Soman, U. G. Characterizing Perception of Prosody in Children with Hearing Loss. (Vanderbilt University, Nashville, Tennessee., 2017).

5. Gordon, R. L., Jacobs, M. S., Schuele, C. M. & Mcauley, J. D. Perspectives on the rhythm-grammar link and its implications for typical and atypical language development. *Ann. N. Y. Acad. Sci.* **1337**, 16–25 (2015).
